# Factors Affecting Polyphenol Biosynthesis in Wild and Field Grown St. John’s Wort (*Hypericum perforatum* L. Hypericaceae/Guttiferae)

**DOI:** 10.3390/molecules14020682

**Published:** 2009-02-11

**Authors:** Renato Bruni, Gianni Sacchetti

**Affiliations:** 1 Dip. di Biologia Evolutiva e Funzionale – Sez. Biologia Vegetale, Viale G. Usberti 11A, I-43100, Università degli Studi di Parma, Italy; 2 Dip. di Biologia ed Evoluzione – Sez. Risorse Agrotecnologiche e Farmaceutiche AgriUnife, C.so Ercole d’Este 32, I-44100, Università degli Studi di Ferrara, Italy

**Keywords:** *Hypericum perforatum*, Hypericins, Polyphenols, Flavonoids, Secondary metabolism optimization

## Abstract

The increasing diffusion of herbal products is posing new questions: why are products so often different in their composition and efficacy? Which approach is more suitable to increase the biochemical productivity of medicinal plants with large-scale, low-cost solutions? Can the phytochemical profile of a medicinal plant be modulated in order to increase the accumulation of its most valuable constituents? Will polyphenol-rich medicinal crops ever be traded as commodities? Providing a proactive answer to such questions is an extremely hard task, due to the large number of variables involved: intraspecific chemodiversity, plant breeding, ontogenetic stage, post-harvest handling, biotic and abiotic factors, to name but a few. An ideal path in this direction should include the definition of optimum pre-harvesting and post-harvesting conditions and the availability of specific Good Agricultural Practices centered on secondary metabolism enhancement. The first steps to be taken are undoubtedly the evaluation and the organization of scattered data regarding the diverse factors involved in the optimization of medicinal plant cultivation, in order to provide an interdisciplinary overview of main possibilities, weaknesses and drawbacks. This review is intended to be a synopsis of the knowledge on this regard focused on *Hypericum perforatum* L. (Hypericaceae/Guttiferae) secondary metabolites of phenolic origin, with the aim to provide a reference and suggest an evolution towards the maximization of St. John's Wort bioactive constituents. Factors considered emerged not only from in-field agronomic results, but also from physiological, genetical, biotic, abiotic and phytochemical data that could be scaled up to the application level. To increase quality for final beneficiaries, growers’ profits and ultimately transform phenolic-rich medicinal crops into commodities, the emerging trend suggests an integrated and synergic approach. Agronomy and genetics will need to develop their breeding strategies taking account of the suggestions of phytochemistry, biochemistry, pharmacognosy and pharmacology, without losing sight of the economic balance of the production.

## Contents

1. Introduction
2. Genetic variability: chemotaxonomy, phenotypic variations and breeding within *Hypericum* genus; a focus on *H. perforatum* L.
3. Ontogenetic stage and seasonal variation
4. Agricultural practices: irrigation, soil, fertilization
5. Biotic and abiotic stress
6. Post-Harvest and stability
7. Conclusive remarks
8. References

## 1. Introduction

The rapid and often unpredictable success of some herbal drugs within the medicinal and health food market is changing several criteria of management for botanical products and is posing challenges in diverse research fields. In fact, plants traditionally collected in the wild and used as traditional medicines by a scanty number of people (usually in a circumscribed area of the world) can nowadays became, within a half-dozen years, valuable sources of best-seller drugs marketed worldwide. Such “local to global” transitions can induce dramatic changes in plant collection, cultivation, supply, handling and marketing practices. In fact, to meet the requests of a wider number of consumers, the demand for raw plant materials usually skyrockets very quickly, making simple plant collection in the wild insufficient and unreliable, heightening a subsequent risk of biodiversity loss and ultimately inducing a shift to wide scale cultivation [1]. Moreover, within the herbal market not just quantity but also quality must be taken into account: the more the consumers, the more the needs for products reliable accounting to their composition, efficacy, safety, toxicity and price. It comes as no surprise, thus, that industrial and commercial sectors of botanical derivatives are constantly pushing towards the standardization of raw materials, with the introduction of GMP (Good Manufacturing Practices) and GAP (Good Agricultural Practices) endorsed yet at the cultivation level, with the aim to reduce costs and consolidate their putative markets [2]. 

Controlled production of medicinal plants appears to be an high priority and can be considered as a key factor towards the “perfect” standardization and to meet the increasingly stringent safety requirements requested by the regulatory agencies [3]. It is highly advisable that the standardization process, in order to obtan reproducible results and products, should start from the choice of appropriate and reproducible conditions of cultivation by means of appropriate studies. Their importance is capital in medicinal plant production, because it could lead to the simultaneous settlement of two main problems at the opposite ends of the herbal products market: i) the maximization of the production and ii) the certainty of high-quality over-the-counter products in terms of efficacy and safety. However, being the quality of natural drugs defined by the amount of specific active principles and being those substances often of phenolic origin (e.g. flavonoids, catechins, anthocyans, anthraquinones, phenylpropanoids), agronomic studies should not be limited to biomass maximization and cost limitation as they were in the past, but also highly focused on phenolic secondary metabolism optimization. The main issue in medicinal plant standardization seems to reside within the intrinsic variability of secondary plant metabolism. On one side, commercial manufacturing seeks and requires standardized processes which are as steadfast and repeatable as possible, but on the other side Nature responds with the instrument that best functioned through evolutionary adaptation: dynamic complexity. What one normally tends to see as a useful, substantially monolithic source of healthy substances is, in reality, an elaborate, plastic, ever-changing, adaptative system of ecological communication, defense and interaction between the plant and its environment. As such, it tends to adapt by changing in response to the stimuli it receives and thus can offer a number of variables one can works on to elicite the production of desired metabolites. Phenols and polyphenols are no exception in this regard.

The complete optimization of agronomic conditions according to phytochemical production, actually, is a long and huge effort, needing years and long term financial support. Factors to be considered should emerge not only from in-field agronomic results, but also from physiological, genetical, biotic, abiotic and phytochemical data that could be scaled up to the application level. Moreover, being such approach financially burdensome, it should be wisely undertaken only on a globally marketed and economically sound plant, like *Hypericum perforatum* surely is.

*Hypericum perforatum* L. (Hypericaceae = Guttiferae) is presently considered as one of the few economic plants that has successfully completed the transition from noxious weed to wild collected resource and then to cultivated crop. Because of its well-established market position, its popularity and efficacy, St. John’s Wort (SJW) is reputed as one of the best-selling herbs of the last decade, despite a recent negative trend: in 1998 its market value exceeded 570 million US$ worldwide and 200 million US$ solely in the USA and several hundreds of cultivated hectares are nowadays available in Europe [3,4,5], where the market request for crude SJW drug in 1999 was above 5,000 t [6]. According to the US and the EC Pharmacopoeias, the crude drug consists of the dried flowering tops or aerial parts of the plant, at present coming almost exclusively from field-grown plants. Drug is used as-is or as an extract both as monopreparation or in multi-ingredient formulations. Three drug qualities are known: *Hyperici herba* (cuttings of the entire plant at flowering, including stem); *Hyperici herba flowering horizon* (cuttings of the upper 30 cm of the entire plant at flowering, including stem) and almost pure flowers. Despite being a perennial plant with a life cycle of 3-5 years, SJW can be intensively cultivated for no more than 2-3 years, with the optimum biomass production at the second (30-160, 170-400, 300-500 g/m^2^ dry herba) [7]. 

**Figure 1:**
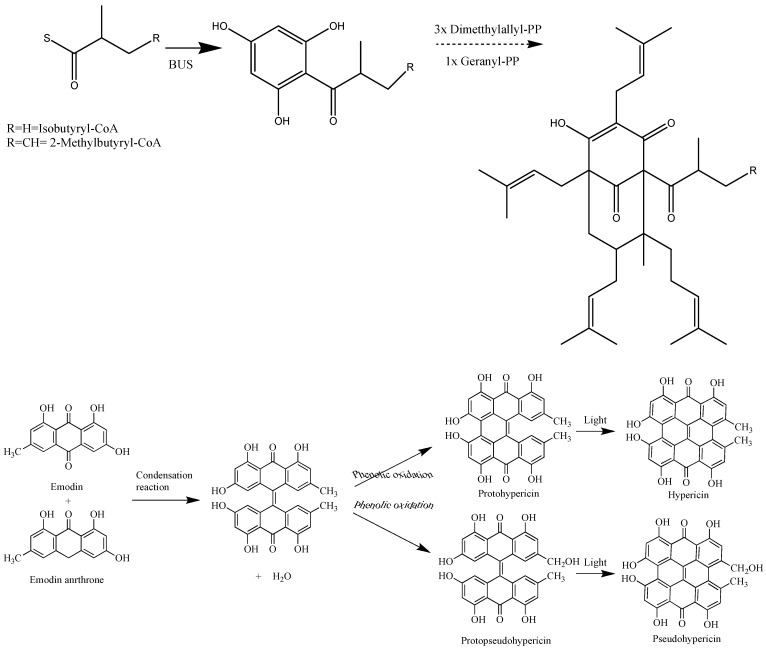
Basic biosynthetic pathway of hyperforins and hypericins.

The bulk of the SJW phytocomplex is characterized by a broad spectrum of secondary metabolites (Table 1) and major constituents encompass mostly polyphenols like naphthodianthrones (hypericin, pseudohypericin and three less abundant – up to 50-fold lower – derivatives, namely protopseudohypericin, cyclopseudohypericin and protohypericin); flavonoids (hyperoside, quercitrin, isoquercitrin, rutin, quercetin); biflavones (amentoflavone, biapigenin); phenylpropanes (chlorogenic acid); acylphloroglucinols (hyperforin, adhyperforin) and an essential oil rich in sesquiterpenes [8]. Aminoacids like glutamin, asparagin, arginin and neurotransmitters like GABA have also been found in noticeable amounts [6], while a melatonin-rich germplasm-line has been selected, albeit only *in vitro* [9]. However, a substantial survey of content and variability of such substances is lacking at present. Distinct biosynthetic pathways are involved in the formation of the phytocomplex and may be differently affected by genetic, environmental and agronomic variations. Some of the aforementioned metabolites can be found in significant amounts only in *H. perforatum*, as in case of hyperforin [10]. In phytomedicine, the whole extract and some defined phytochemicals are reputedly responsible or co-responsible for a plethora of pharmacological properties, ranging from wound healing and antiseptic, to antiviral, anti-AIDS *in vitro* replication, anti-inflammatory, anti-carcinogenic, ethanol intake inhibition, photosensitization, inhibition of cytochrome P450 enzymes, apoptosis-inducing activities, anxiolytic and antiatherogenic [11,12,13,14,15,16,17,18,19,20,21,22,23,24]. The most significant and adequately validated use, however, is in the symptomatic treatment of mild-to-moderate depression for which SJW efficacy has been validated through a number of clinical trials and meta-analytical studies, and recently good perspectives emerged also in the field of major depression [25,26]. 

**Table 1:** Localization and indicative amounts of most significant constituents of *H. perforatum* phytocomplex.

Substance	Approximate amount (mg/g dry weight)
**Naphtodianthrones**	
Hypericin	0.1-7^a^
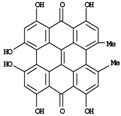	
Pseudohypericin	0.1-12^a^
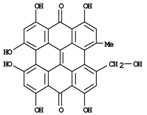	
**Localization**	^a^Dark glands in leaf and petal margin; stamens
**Acylphloroglucinols**	
Hyperforin	0.3-150^b^
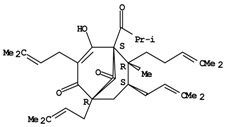	

**Localization**	^b^Translucent glands in leaves, carpellar leaves and sepals
**Flavonoids**	
Hyperoside	1-25^c^
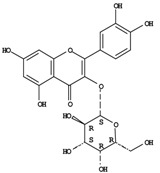	
Rutin	0-35^c^
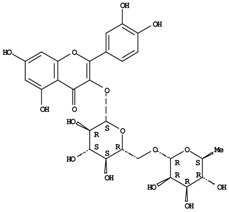	
**Localization**	^c^Floral dehiscent leaves: sepals, stamens, petals. Likely accumulation in vacuoles.
**Biflavones**	
Amentoflavone	0-1.8^d^
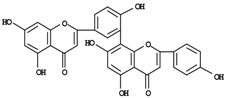	
Biapigenin	0.3-10.2^d^
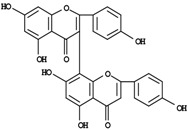	
**Localization**	^d^Floral deiscent leaves: sepals, stamens, petals. Likely accumulation in vacuoles.
	

^a,b,c,d:^ same letters indicate sites of maximum accumulation; amounts referred to reproductive parts.

Typically, the hydroalcoholic extract is prepared and standardized for 2.4-3.2 mg/g dry weight hypericin, a naphtodianthrone reputed as the main quality marker, but the extract contains all the above mentioned phenolic substances, most of them exerting to some degree a direct or indirect antidepressant activity [27,28,29,30]. Such complexity poses troubles in phytochemical standardization and definition of quality by analytical means. In particular, the major issue lies in the choice of an adequate marker: i.e., while Pharmacopieia Helvetica 8 suggest the tracking of rutin, Deutscher Arzneimittel-Codex 79 more properly suggest the choice of hypericin as a reference substance.

**Figure 2:**
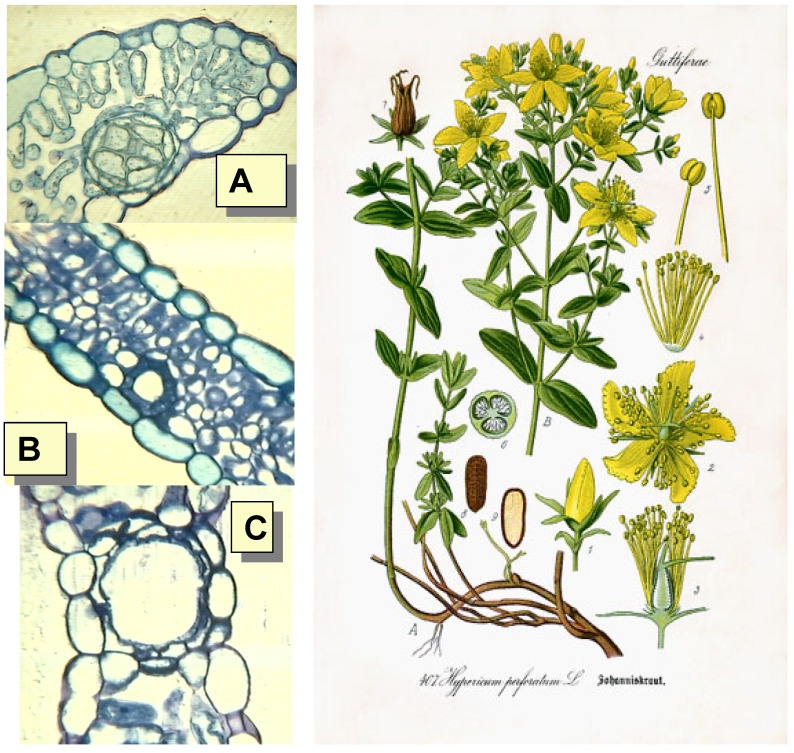
*H. perforatum* from Prof. Dr. Thomé, Otto Wilhelm, Flora von Deutschland, Österreich und der Schweiz; A) Black nodule from *H. perforatum* leaves, B) Leaf canal; Translucent cavity (Courtesy Prof. A. Bianchi, University of Parma, Italy).

Pharmacopoeia Europaea and United States Pharmacopoeia have recently proposed a three-way standardization, suggesting to simultaneously quantify hypericins, hyperforins and flavone glycosides [31]. This is, however, a problem that only a wider employ of metabolomic techniques like NMR will solve in the foreseeable future [2]. Since the purported health effects and market value of *hyperici herba* are strictly related to phytochemical profile, hypericins are, at present, the most touted commercial markers of *H. perforatum* drug value and producers may receive $2,000 to $3,000 more per acre if their crops are hypericin-rich [32]. As a consequence, great effort has been directed towards the management of field cultivation, in order to maximize both gross yield and quantity of phytochemicals reputed responsible for the plant’s therapeutic properties [33]. According to the perspectives and the contingent needs of *H. perforatum* market (e.g., in Europe, most commonly cultivated varieties “Topas”, “Anthos”, “Elixir”, “New Stem” do not always fulfill the above mentioned qualitative and quantitative market requirements), the main objective of this review is to summarize data treating topics directly and indirectly related with factors influencing the production of biomass and polyphenolic secondary metabolites of *Hypericum perforatum* L*.*. The aim is to provide an updated reference on the state-of-the-art of its cultivation and to suggest its evolution towards the maximization of phenolic bioactive constituents. 

## 2. Genetic variability: chemotaxonomy, phenotypic variations and breeding within *Hypericum* genus; a focus on *H. perforatum* L.

### 2.1. Hypericum genus: chemodiversity

The genus *Hypericum* is widely distributed through the temperate regions and is represented by approximately 450 species, of which only a limited number is known to contain hypericins. Hypericin and related anthrone derivatives are genera-specific compounds, not detected in the rest of the *Guttiferae* family, nor elsewhere in the Plant Kingdom. Chemotaxonomic studies on the genus have been initially summarized by Mathis and Ourisson [34] regarding hypericin, terpenes and unsaponifiable content. Such a massive, although somewhat outdated effort, evidenced the presence of hypericins mainly in the sections *Euhypericum* and *Campylosporus* and in general the species characterized by the major presence of red glands were found to contain also the higher amounts of hypericins. Recently, an extensive survey on 74 *Hypericum* taxa (approx. 20% of the entire genus) from different continents has been conducted, evidencing and confirming a consistent chemodiversity between Sections of the genus [35]. Hyperforin was detected only in few species belonging to the Sections *Hypericum, Myriandra, Ascyreia*, while hypericins were detected only in species of the Sections *Hypericum, Adenotras, Drosocarpium* and no chemotaxonomic relevance was found for those polyphenols. Unfortunately, however, the paper did not provide quantitative, but solely qualitative results. Within the genus, hypericin and pseudohypericin are absent or negligible in the primitive sections and the latter have been found only in some *Hypericum* species. In some taxa (*H. hirsutum, H. empetrifolium*) only hypericin can be found [36]. According to the quantitative data provided by Kitanov, the presence of hypericins is relevant for chemotaxonomy and those polyphenols seem to be specific only for taxa of more phylogenetically advanced sections of the genus (*Hypericum, Drosocarpium, Thasia, Adenosepalum*), depicting themselves as an evolutive factor introduced through adaptation. Such characteristic is also confirmed by the wide range exhibited by *H. perforatum* on its ecological adaptation scale [37]. Infrageneric chemotaxonomic surveys, moreover, provide suggestions on hypericins-rich Hypericum species: i.e *H. boissieri, H. barbatum, H. rumeliacum* may contain a 2-4 fold higher amount of hypericins than *H. perforatum*, but only a small part of the genus has been screened and approx ¾ of *Hypericum* species have not been surveyed for their hypericin/hyperforin content. Due to evident advantages (cultivation, wolrdwide distribution) *H. perforatum* is at present the sole source of *Hyperici Herba*. 

### 2.2. Hypericum perforatum: chemodiversity in the wild

Among the *Hypericum* genus, the therapeutic use and the commercial value of *H. perforatum* are absolutely pre-eminent, as a consequence of the favourable combination of high hypericin content, wide geographical diffusion and deep ethnopharmacologic history. This species, considered native to Europe, Middle East and Norther Africa, has been progressively introduced (both casually and purposedly) to most temperate and tropical mountain regions of the entire planet over a century ago and thus adapted itself to different climatic and ecological conditions, often reaching weed status. SJW is frost-resistant, chalk-loving, light-loving and thrives in disturbed habitats like roadsides, grazing lands and pastures and is also touted as a potentially noxious weed. Sometimes such widespread distribution contributed to the development of local cultivars and/or subspecies, which may differ for their gross biomass yield and secondary metabolite production. Variations may be related to environmental conditions that may vary widely from region to region or from the genetic variation that may occur among regionally distinct groups after decades of isolation (up to four subspecies of SJW have been recognized so far). Such reservoir of natural variability may serve as a sound basis for breeding development but is, at the same time, the main reason of the high chemodiversity of SJW. Phytochemical investigations carried out on different varieties and accessions of *H. perforatum* in fact rarely provide homogeneous results, both regarding phloroglucinols and naphtodianthrones. Intraspecific chemodiversity is seemingly present also under controlled conditions even in the early stages of plant development [38]. From an evolutive standpoint, such behavior can be considered characteristic of a very ecologically aggressive species.

Examples of such diversity for hyperforin, hypericin and pseudohypericin are summarized in Table 2 and these observations are worth further consideration. For example, some authors reported an interesting qualitative variation between two *H. perforatum* subspecies (ssp. *perforatum* and ssp. *veronense,* a Siberian native taxa with small ovato-elliptical leaves), with the latter being devoid of both hypericins and hyperforin [35]. Such evidence could suggest a wider and more accurate phytochemical screening of *H. perforatum* subspecies, in order to exclude those that are unsuitable for the herbal market or detect those better suitable for the breeding of polyphenol-rich lines. Moreover, consistent variations (above 200%) were found according to hypericin content between different germplasm lines grown in the same habitat conditions [39]. Furthermore, different accessions can provide variations exceeding 100% in fresh and dry mass of the aerial parts (at reproductive stage). As regarding the hyperforin biosynthesis, great intraspecific variation (up to four-fold, from 3 to 1.3 mg/g dry weight) was observed in seedlings [38]. 

Most preminent morphologic intraspecific difference within *H. perforatum* is the presence of broad leaved (*H. perforatum* var. *perforatum*, originary of Northern Europe) and narrow leaved varieties (*H. perforatum* ssp. *angustifolium*, originary of Southern Europe but extensively found in Australia [40]. The morphological distinction can be made via the evaluation of leaf length/width ratio, which is 3.1 for narrow and 2.0 for broad. Varieties with intermediate or small leaves are also known (*H. perforatum* ssp. *microphyllum;* ssp. *veronense*) [41]. When it comes to polyphenol content, hypericin and leaf glands density of narrowleaf variety has been often noticed to accumulate up to 4 times higher than that of the broad-leaf [40,41]. Recently, ssp. *perforatum* was described as a richer source of than ssp. *angustifolium*, which on the contrary provided higher amounts of hypericins and was devoid of rutin [42]. According to some reports, ssp. *angustifolium* is mainly diploid and biannual and is characterized by early flowering capacity, low biomass productivity and high degree of generativity, while ssp. *perforatum* and ssp. *latifolium* are mainly polyploid, easier to adapt to different ecological conditions and endowed of higher productivity [43].

**Table 2:** Amount of hyperforin (Hpf), hypericin (Hyp) and pseudohypericin (Pse) in vegetative and reproductive parts of wild and cultivated *H.perforatum* and related cultivars of different geographic origin.

Origin/Cultivar	Amount^1^			Method	Notes	Ref.
	Hpf^2^	Hyp^3^	Pse^4^			
**North American **						
Wild - Canada, BC	nm^5^	0.33-0.63	nm	HPLC	Aerial parts at full flowering	[52]
Wild - Canada, Nova Scotia	nm	0.12-0.29	nm	HPLC	Aerial parts at full flowering	[52]
Wild - Canada, Ontario	nm	0.13-0.27	nm	HPLC	Aerial parts at full flowering	[52]
Wild - Canada, Oregon	nm	0.24-0.54	nm	HPLC	Aerial parts at full flowering	[52]
Wild - USA, California	nm	0.11-0.44	2.78-4.27	HPLC	Aerial parts Higher values in flowers	[50,51]
Wild - USA, Montana	nm	0.05-0.18	0.76-1.07	HPLC	Aerial parts Higher values in flowers	[50,51]
Wild - USA, Oregon	nm	0.37-3.87*	nm	UV^6^	Aerial parts * Flowers only	[49]
Wild - USA, Oregon	nm	0.60 mg/g	2.86 mg/g	HPLC	Aerial parts Higher values in flowers	[50,51]
Cultivated – USA	*27.37 **14.01	*1.46 **0.38	*2.51 **0.71	HPLC	*Flowers ** Leaves	[114]
**South American **						
Cultivated – Brazil	nm	3.83	nm	HPLC	Leaves, stems, vegetative stage	[150]
Cultivated - Chile	nm	1.1-1.2^8^	nm	UV	Aerial parts at full flowering	[171]
Cultivated - Chile	nm	1.9-2.5	nm	UV	Flowers	[53]
**European and Middle East **						
Wild - Armenia	150	0.23-0.05	nm	HPLC	Low variability of samples collected from different sites. Unspecified aerial parts	[65]
Wild - Armenia	nm	0.06-0.64	0.2-1.6	HPLC	FlowersFrom 10 different regions	[66]
Wild - Bulgaria	nm	1.25	nm	UV	Unspecified aerial parts	[36]
Wild - Croatiassp. perforatum	nm	0.3	nm	HPLC	Unspecified aerial parts. Richer in flavonoids	[42]
Wild - Croatia,ssp. agustifolium	nm	1	nm	HPLC	Unspecified aerial parts. Devoid of rutin	[42]
Wild - Greece (Crete Island)	nm	*1.1 **0.25	*1.3 **0.17	HPLC	*Flowers **Leaves and petioles	[67]
Wild - India	1.66-4.62	1.19-2.68	13.74-41.64	HPLC	Aerial parts	[72]
Wild - Iran		1.01	nm	UV	Aerial parts af full flowering	[168]
Wild - Italy	*12.4-21.1 **31.5-41.5	*2.8-6.6 **0.6-2.9	*5.2-10.8 **2.4-6.2	HPLC	*Dried flowers only **Fruits. Samples from 5 different italian regions	[58]
Wild - Italy	8.3-31.81	8.34-14.79	nm	HPLC	Aeria parts at full flowering ssp. *angustifolium* and ssp. *perforatum* evidenced both genetical and ecological differences	[60]
Wild – Italy	*10.72-23.32 **14.55-24.26	*0.35-0.52 **1.12-1.72	nm	HPLC	* ssp. Perforatum ** ssp. Veronense Aerial parts at full flowering	[173]
Wild - Italy (Tuscany)	0.84-4.82	0.02-0.11	0.03-0.33	HPLC	Flowers only. Chemotype devoid of rutin	[57]
Wild - Italy (Tuscany)	82.5-52 101.4-109.5 fw^ 7^	*0.03-0.2	nm	HPLC	Flowering tops *Expressed as total naphtodianthrones	[151]
Wild - Russia (Udmurtia)	nm	12 fw	nm	UV	Flower buds	[63]
Wild - Serbia	3.55	0.17	nm	HPLC	Aerial parts at full flowering	[64]
Wild - Serbia	1.52	0.2	0.5	HPLC	Aerial parts at full flowering	[172]
Wild - Solvenia	13.59	5.20	5.22	HPLC	Aerial parts at full flowering	[55]
Wild - Turkey	nm	*0-**2.73	nm	HPLC	* Stems ** Flowers	[100]
Wild - Turkey	nm	0.44-2.82	nm	HPLC	Aerial parts at full flowering	[62]
Wild - Turkey	nm	2.15-2.46	nm	UV	Aerial parts at full flowering	[93]
Wild - Turkey	nm	0.28-4.46	nm	UV	Aerial parts at full flowering	[61]
Cultivated - Denmark	3 fw^7^	nm	nm	HPLC	Leaves only	[92]
Cultivated - Germany	2	0.1-5.2	0.1-16	HPLC	Flowering tops	[6]
Cultivated - Germany cv. Hyperiflor	12.3	0.8	nm	HPLC	Aerial parts af full flowering	[136]
Cultivated - Germany cv. Hyperimed	26.51	0.87	nm	HPLC	Aerial parts at full flowering	[136]
Cultivated - Germany cv. Topaz	12.81	1.11	nm	HPLC	Aerial parts at full flowering	[136]
Cultivated - Hungary	nm	0.10-2.64^8^	nm	HPLC	Aerial parts at full flowering Selected breeding lines.	[7]
Cultivated - Hungary cv Topas and selected breeding lines	nm	0.64-3.88	3.18-11.88	HPLC	Aerial parts at full flowering	[43]
Cultivated - HungaryHybrids selection	nm	1.52-11.9 fw^7^	nm	UV	Reproductive parts.	[68]
Cultivated - Italy cv. Zorzi	nm	1.36	nm	HPLC	Aerial parts at full flowering	[139]
Cultivated - Lithuania	nm	0.23-1.24	nm	HPLC	Flowering tops Seeds from 21 wild accessions	[54]
Cultivated - Poland	nm	7-9	nm	HPLC	Aerial parts at full flowering	[169]
Cultivated - Slovakia	nm	1.59	nm	UV	Aerial parts at full flowering 4th generation of selected ex-vitro plants from regenerated in vitro somaclones	[48]
Cultivated - Slovakia	*91.6-107.5	**1.1-5.8	***0.7-6.9	HPLC	*Isolated unripe fruits **Buds Fluctuation during ontogenesis Samples devoid of rutin	[94]
Cultivated - Switzerland	*0.9-6.4	**0.9-6.1	nm	HPLC	*Maximum in flowers with open petals **Maximum in closed buds	[170]
Cultivated - Switzerland	nm	0.23-1.72	0.81-3.27	HPLC	Aerial parts at full flowering Screening of 24 accessions	[69]
Cultivated -Switzerland	nm	0.34-1.08	1.25-2.36	HPLC	Aerial parts at full flowering cv Topas, Hyperimed, Elixir	[69]
**Oceania **						
Wild - Australia	nm	0.12-0.8	nm	HPLC	Aerial parts at full flowering	[52]
Wild - Australia	nm	1,02-4.84	nm	UV	Leaves only	[71]
Wild - Australia	nm	*1.2-2.35 **2.97-5.02	nm	UV	* Broadleaved ** Narrowleaved Aerial parts at full flowering	[41]
**Greenhouse or controlled environment**						
Controlled Environment	*3.13-7.39 fw **8.46-13.91 fw	*0.03-0.09 fw **0.75-1.18 fw	*0.05-0.16 fw **0.98-1.47 fw	HPLC	*Shoots **Flower buds	[39]
Controlled Environment	30	1.3	2.8	HPLC	Leaves	[112]
Controlled Environment	nm	18-38	42-115	HPLC	Isolated flowers	[89]
Greenhouse Cultivated	*0.65-3.17 **1.97-9.00	*0.57-3.93 **2.31-6.72	*1.07-4.10 **1.13-3.97	HPLC	* Flowering top ** Flowers. Seeds wild-collected in France and Switzerland.	[59]
Greenhouse Cultivated cv. Anthos	5.09	0.09	0.11	HPLC	Unspecified aerial parts	[65]
Greenhouse Cultivated cv. New Stem	3.4	0.09	0.11	HPLC	Unspecified aerial parts	[65]
Greenhouse Cultivated cv Topaz	2.92	0.06	0.11	HPLC	Unspecified aerial parts	[65]
Greenhouse Cultivated cv. Topaz	*1.89 **4.45	*0.82-2.10 **1.31-5.09	nm	HPLC	* Flowering top ** Flowers Seeds wild-collected in France and Switzerland.	[59]
Greenhouse Cultivated cv. Topaz	nm	*0.23-0.39 **0.29-0.59	*0.44-0.89 **0.71-1.52	HPLC	*5 cm tops ** Flowers only	[96]

^1^ All data are in mg/g dry weight, othervise differently stated; ^2 ^Hpf – Hyperforin; ^3^ Hyp- Hypericin; ^4^ Pse – Pseudohypericin; ^5 ^Not measured;^ 6^ UV-Spectrophotometric determinations at 590 nm may suffer from interference from other pigments noticed by some authors. As a result such methodology may provide higher values than HPLC quantifications and no distinction between hypericin and pseudohypericins is possible; ^7^ Fresh weight; ^8^ dryness of the sample not specified.

To create an even more intricated scenario, SJW seems to offer a typical example of chemical polymorphism, as different metabolic behaviors have been detected between plants growing side by side. This could be also a consequence of the prevailing apomictic reproduction of SJW or of a possible liability to hybridization [44,45]. Ploidy has been evaluated and found to be indirectly affecting hypericin content. The alteration of ploidy from tetraploid and diploid to pentaploid induced an increase in biomass production, but did not change the biosynthetic rate of polyphenols and hypericins, thus leading to a “dilution” of those metabolites and a lower yield of extraction. Diploid populations, which are obligate sexuals, are usually significantly richer in hypericins than tetraploid, which are facultative apomicts [38,46,47,48]. 

Wild populations of *H. perforatum* have been evaluated in various countries and continents. Data presented, however, are quite discrepant due to phenotypic variations but also to different analytical approaches, blossoming stages of collection, extraction methods and thus are often hardly comparable (Table 2)

#### 2.2.1. Chemodiversity – American populations

Two papers have surveyed the hypericin content of various populations from northwestern USA (California, Oregon, Montana), detecting a higher red glands density and content of hypericins in Californian samples and a lower one in Montana and Oregon accessions [49,50]. An analogous report from the same geographical zones provided similar mean values, although evidencing a greater range of variation [51]. Mean floral concentration of pseudohypericin and hypericin were higher during anthesis (July, August) while in leaves a maximum was reached in August. Similar observations were obtained for leaf and flower number. Hypericin concentrations from whole plants collected in Nova Scotia (Canada) were also comparable, leading to suggest that, at present time, wild north American populations seem to be rather uniform in terms of phytochemical profile, despite the fact that westernmost ones (British Columbia, California, Oregon) seem to be slightly richer in hypericins than those of the eastern regions (Ontario, Nova Scotia) [52]. Limited studies on South American accessions performed a comparison between Chilean wildtype SJW and plants grown from seeds of European origin, with the latter always performing better both in terms of biomass yield and phytochemical content [53].

#### 2.2.2. Chemodiversity – European and Middle East populations

A large number of reports is available on the different availability and distribution of polyphenols in European accessions. Radusiene and Bagdonaite [37] noticed, for wild Lithuanian populations at full bloom, better results in terms of flavonoidic content than those obtained from the locally cultivated cultivar “Zolotodolinskaja”. The correlation between phytochemical content and morphological traits evidenced a relationship between flavonoidic content and leaves length. A more detailed study on this regard found a relationship between morphological traits and quali-quantitative characteristics of the drug, evidencing that wild Lithuanian accessions with black glands both on petals and pistil provide a better quality drug in terms of hypericin abundance and gross yield and could thus offer a viable genetic pool for breeding purposes [54]. In the same work, samples were found to be richer in rutin if compared to other European collections. Regarding biomass production, authors evidenced significant differences in most phenotypic traits, leading to the individuation of two different groups in Lithuanian wild population of SJW. Other authors have evaluated wild Hungarian populations, detecting a high variability in hypericin content but a rather stable flavonoid pattern, with the sole exception of rutin, which ranged from 0.08 to 24 mg/g dry weight [43]. The populations rich in rutin are usually low in hyperoside and *vice-versa*. Thus two main chemotypes were described: one with hyperoside and isoquercitrin and one with rutin and hyperoside in equal amounts, while the combination of a good amount of both hypericin and flavonoids was not noticed. Umek *et al*. [55] screened a few Slovenian populations of SJW noticing that rutin and hyperoside and in general flavonoidic content correlate positively with altitude. Similar reports have been reported for populations in nearby Austria, where lower amounts of hypericins were detected, however [56]. Three commercial cultivars (Topas, Hyperimed, Elixir) and 21 wild accessions were evaluated and Topas was found to be the worst performer in terms of flavonoid and hypericin content, while a wild genotype was found to be particularly suitable for commercial exploitation, being 26% richer in flavonoids and 79% richer in hypericins [5]. Regarding rutin, it must be mentioned that an extensive screening of Italian chemotypes detected a chemotype without rutin in which hyperforin is higher than usual and thus may represent an interesting starting point for breeding [57]. At the same time, Buter *et al*. [33] individuated two interesting accessions rich in rutin or in hypericin. This is relevant as some reports have however recently outlined that rutin-low extracts have no effect in antidepressant pharmacological experiments performed *in vivo* [28]. In Italy, a geographical screening has been also conducted. The crude drug collected at fruit ripening stage had the highest content in phloroglucinols and the lowest level of both naphtodianthrones and flavonols. Phloroglucinols peaked in samples collected in Puglia followed by Lombardia and Veneto regions, while hypericins and flavonols were highest in samples harvested in Sardegna and Trentino [58]. Noteworthy, the same authors noticed a strong degree of correlation between hyperforin increase and hypericins decrease, but previous papers report the absence of significant correlations between hypericin content and other contituents of SJW phytocomplex [33,59]. The lack of correlation could be also a consequence of the different sites of biosynthesis and accumulation for such substances, as described later. Looking at the quantitative aspects, there are some important variations that surely depends on the collection site and ecological factors. It is noteworthy to observe that Italian *H. perforatum* ssp. *angustifolium* and ssp*. perforatum* showed highest values of hyperforin when collected in different sites, while two distinct accessions of ssp. *angustifolium* provided different results both in terms of hyperforin and hypericin content. The accessions particularly rich in hypericin were conversely lower in hyperforin [60].

Within 39 accessions from France and Switzerland evaluated for phenolic content driven breed selection during a two-year span, a strong interaccession variability was noticed. Hyperforin levels ranged from 0.65 to 3.17 mg/g dry weight in flowering tops and from 1.97 to 9.0 mg/g dry weight in flowers, while hypericins were comprised between 0.7 and 3.9 mg/g dry weight in flowering top and between 1.6 and 8.13 mg/g dry weight in flowers [59]. Forty-five populations collected in geographically distinct Turkish regions bordering the Black Sea provided hypericins content ranging from 0.28 and 4.46 mg/g dry matter. Intra- and inter-regional variations were detected [61]. The authors concluded that despite the high ecological differences between collection sites, genetic factors were more responsible than phenotypic variation for different hypericin content. In a similar survey 10 accessions from sites in Northern Turkey (presenting different climatic and geographic conditions) were evaluated for phytochemical and morphological variability and significant differences were detected in hypericin, chlorogenic acid, rutin, hyperoside and quercitrin content [62]. On the contrary, quercetin and apigenin-7-*O*-glucoside were constant and an extremely high amount of hyperoside (up to 22.28 mg/g dry weight) was detected. Samples collected in the Russian republic of Udmurtia were found to contain 12 mg/g fresh weight of hypericin in flower buds [63]. Balcanic accessions provided good hyperforin content, as did samples collected in Armenia, for which hyperforin was detected in concentrations over 150 mg/g dry weight, the highest recorded worldwide [64,65]. Samples were also characterized by a limited variability. However, in a previous report samples collected in different Armenian regions gave less striking results, comprised within 0.2 and 1.6 mg/g dry weight for pseudohypericin and 0.06 and 0.64 mg/g dry weight for hypericin [66]. Floral parts of Cretan populations were found to have a mean hypericin content of 1.3 mg/g dry weight and similar amounts of precursors protohypericin and protopseudohypericin [67].

In Europe some breeding experiments have been undertaken and have provided interesting results both in terms of biomass production and selection of lines with enhanced polyphenol content [6]. Within 153 lines selected and screened, a multiple trend and three groups were observed: 1) a group with high rutin, average flavonoid and low hypericin content. These characteristics seems to be the most frequently encountered; 2) a group with extremely low rutin and high amounts of hyperoside, flavonoids and hypericins. This group provided the highest hypericin production; 3) a cluster with a double amount of hypericin if compared to the first group, but provided with higher levels of biapigenin and hyperforin. A similar work was made in Hungary starting from 18 populations and evidencing three different patterns in hypericin secretion during a three-year cultivation: 1) a group producing high amounts of hypericins only during the second cultivation year, 2) a group with low production in the second year and 3) a cluster characterized by a constant hypericin biosynthesis during the three-year span [43]. A hyperoside-rich (10-17 mg/g dry weight) line was obtained through selection and selected lines always exceeded their parents in phytochemical content. Similarly, selection enabled the production of more than 400 g/m^2^ of *Hyperici herba*, ¼ of which made of flowers, during the third cultivation year. The overall results of such researches seem to converge on the definition of two main kinds of behavior: one constituted by plants that provide a quite constant phenotypic response and offering a possibly genetically determined properties and one with a more variable habit that is prone to provide major modifications according to changed environmental factors. Starting breeding from accessions provided with an optimum phytochemical profile, suitable for specific phytotherapic applications and/or productive needs (i.e., high amount of hypericins, high or low amount of hyperforin and rutin) is thus possible.

Regarding the possible biotechnological development of field-grown plants, somaclones obtained *in vitro* and their four successive seed-derived *ex-vitro* generations were evaluated and bred to increase hypericin content, evidencing the possibility to enhance specific polyphenol content, which resulted increased seven times after *ex-vivo* selection [48]. Such report is also important because confirmed that the level of hypericins is steadily transmitted to the seed progeny and seed propagation is thus suitable to breed high-yielding hypericin lines.

Recently, still under European climates, some effort has been also made to set up hybrids suitable for herbal production of St. John Wort and try to avoid both endemic susceptibility to wilt disease and low hypericin content. Albeit preliminary, these studies showed in progenies a tendency to retain the mother’s properties regarding hypericin content, thus no increase on secondary metabolite content has been achieved in this way [68]. The agronomic selection led however to the breeding of a variety tolerant towards the aggression of *Colletotrichum* antrachnose, one of the most noxious phytopathies affecting cultivated SJW, providing at the same time a good hypericin content (up to 2.84 mg/g dry weight) [69]. Regarding cultivars already employed at agronomic level, comparison has been made but some contradictory results emerged. According to Kirakosyan *et al*. New Stem cv. offers the highest total hypericins content and Elixir cv is richer in hyperforin, while according to Richter this cultivar was purported to be rich in hypericins [65,70]. Most of data available on this regard emerge from indirect comparison of distinct research and cannot be unequivocally reputed as benchmark due to experimental differences.

#### 2.2.3. Chemodiversity – Asiatic and Oceanian populations

Phytochemical surveys of *Hypericum perforatum* populations introduced in Asia and Oceania are quite scarce and often outdated [41,52]. Data obtained once again evidenced a broad range of profiles, with higher amounts of hypericins (up to 75%) found in the narrowleaf varieties [71]. Values were consistent with those reported in Europe and the US, e.g. with populations of the Pacific Coast [52]. A recent survey of Indian samples evidenced a great variability in terms of flavonoid, hyperforin and hypericin content, with some accessions providing interesting profiles. Hyperforin, in particular, ranged from 3.8 to 24.6 mg/g dry weight and hypericins from 2.9 to 5.3 mg/g dry weight. A non conclusive and partial correlation between genetic and phytochemical profiles of the wild collected material was found [72].

#### 2.2.4. Future developments

A large range of variability - up to 200% for pseudohypericin and to 55% for hypericin content in some cases - within SJW germplasm has been observed worldwide, both in wild and in cultivated populations and the same was observed also for fresh and dry mass production, for which were recorded variations above 100% even if grown under a controlled environment [39]. As it can be easily deduced from this survey, an exhaustive phytochemical characterization of wild *H. perforatum* phenolic chemodiversity is long to be completed. 

A number of challenges exist in bringing a plant from a wild-harvested status to one in which varietal improvement and standardized cultivation practices can improve yields. Selection of diverse populations, varieties or cultivars aimed to the obtention of improved phytochemical profiles seems to be crucial to identify superior germplasm. Thus, in the quest for an elite germplasm for commercial purposes, the screening of biodiversity, the choice of varieties, chemotypes and cultivars play a pivotal role on the evolution of phenotypic response, especially when secondary metabolism in concerned. Under such perspective, data collected during the last decades are conspicuous but unfortunately not conclusive nor adequately organized. Despite being extremely relevant, the evaluation and subsequent selection of *H. perforatum* chemodiversity is in fact scattered and often conducted without taking account of key details. For example, a clear specification of the variety or cultivar evaluated is not always provided in the literature, making the results unfortunately prone to misinterpretations and misleading results. A suitable characterization of accessions with genetic markers would be highly valuable; various genetic fingerprinting methods are becoming available and their widespread use should be endorsed [73,74,75,76,77,78,79,80]. Moreover, careful and detailed description of the habitat of collection for wild accessions would be extremely useful to adequately interpret data obtained, but unfortunately is rarely provided. As we will point out later, stage of plant development, proportion of plant tissues analyzed, time of collection, drying and storage are crucial for hypericin, hyperforin and overall polyphenolic content. An higher degree of uniformity must be encouraged on this regard. Similar considerations can be drawn regarding the choice of the analytical method, as the choice of different UV or HPLC methods of quantitation can translate into results difficult to compare properly.

However, some overall considerations can be drawn out regarding the different polyphenol biosynthesis: the flavonoidic content seems to be weakly influenced by geographical origin and appear as a factor more closely related to the genotype. From a breeder perspective, it remains to be seen if the diversity in hypericin content is a constant and heritable phenomena or simply a phenotypic consequence of ecologic adaptation. On the contrary, hypericin appears to be more correlated to the local conditions of growth. It is known that the variability of a species can increase in relation to its expansiveness across a geographically diverse area and this is typically found in SJW, suggesting that its regional distribution may be an important source of variation and should be considered along with harvest time optimization and processing. Regarding the agronomic selection of peculiar chemotypes, besides the phytochemical differences already mentioned, the amounts of hyperforin and hypericins do not seem to be directly correlated and this may lead to the possibility to develop a specific selection of the SJW germplasm towards cultivars or varieties rich in a specific component. Whether the specific levels of phytochemicals in the studied populations are the result of environmental rather than genetic influences will undoubtedly need further wide-scale field experiments, in which different SJW germplasm is grown for adequate comparison under the same agronomic, environmental and post-harvest conditions. Once the ideal characteristics are obtained, techniques for preservation and propagation of genetically uniform plant material are already available. Germplasm conservation, for example, seems to be possible by cryopreservation without affecting the hypericin content in regenerated populations and micropropagation as well did not affect both flavonoids and hypericins content when *ex-vitro* rooted plants were compared to greenhouse seed-grown individuals [81,82]. 

Chemodiversity survey and traditional breeding seem to be at present the sole viable options for SJW germplasm improvement. In fact, metabolic engineering and the subsequent obtention of transgenically-enhanced *Hypericum perforatum* populations are made difficult by the recalcitrance shown by this plant towards traditional *Agrobacterium*-mediated transformation [83]. The status of the progress in biotechnological production of SJW secondary metabolites has been recently summarized evidencing a higher yeld from *in vitro* production of hypericins and hyperforin and a wider range of manipulating tools, but also higher costs and a recollection of data that can be only seldom scaled up and translated into applicative agronomic strategies [84].

## 3. Phenolic content during ontogenetic stage and seasonal variation

Several studies were performed during the last decade regarding the histochemical and biosynthetic development of leaf nodules and their consequences on the abundance and specific accumulation of SJW polyphenols. *H. perforatum*, in fact, possess an almost unique secretive system, composed by structures of three kinds: canals, translucent essential oil-bearing schizogenous cavities and black multicellular nodules located on the leaf surface, in which hypericins are produced and stored [85]. *H. perforatum* nodules are not present in the cotyledons and do not conform to any internal secretory structure elsewhere described and, the absence of a lumen or any cavity being the most remarkable uniqueness [86]. Moreover, the living cells involved in nodules initially act as a secretory tissue, then lose their functionality and became dead storage areas for secreted phenolic substances. At the end of the reproductive stage, they shrink and lose their shape [87]. Such glands occur in proximity of the leaf and petal margin and are included between the lower epidermis and the palisade tissue and are usually overlapped by outer flat cells probably operating as light filters. Hypericin, in fact, needs photoactivation to became toxic and different polyphenols like flavonoids, tannins and anthocyans are accumulated in outer flat cells likely with the purpose to reduce the light exposure of nodules and hence prevent autotoxicity. Their full development in size occurs 7-10 days after leaf sprouting. Such behavior and the localization in the outer part of the leaf surface seems to confirm a suggested ecological purpose of these polyphenols as a defense against grazing herbivores and fungal infections [88]. 

Various authors have tried, with contradictory results, to establish a correlation between the number and size of glands and the phenolic content [87]. As far as is known, the glands size peaks in leaves after 6 weeks of plant growth (at 8.2 cm^-2^) and each gland seem to contain the same amount of hypericins and of their precursor, the trihydroxyanthraquinone emodin [89]. The number of nodules, however, is seemingly predetermined at the time secretory cells differentiate in the meristem and is reputed fixed [90]. Also *in vitro* the number of dark glands was found to be unchanged between lines differing greatly in hypericin content [65]. Relationships between chemical constituents and gross tissue morphology revealed to be hard to define. This is also confirmed by considerations regarding the putative relationship between red/dark glands and hypericin content. In fact, if the number of secretive structures is predetermined at meristematic level and hypericin can instead vary, then hypericin content within each gland during the plants’ life can vary, thus indicating that this variation is related to the environment and to ontogenetic factors. Therefore, it can be only speculated that higher dark gland density on plant tissue means higher hypericin availability. The presence of glands, however does not seem to be mandatory for hypericin biosynthesis and various evidences are available that hypericins can be massively biosynthesized also in non-differentiated cultured cells on appropriate photoperiod conditions and then stored in vacuoles [91]. Although some reliable histological information is available on hypericin and pseudohypericin biosynthesis, the scenario is less definite regarding hyperforin, as only recently the cellular site of its production and accumulation was defined. Hyperforin, despite the production of its core via a polyketid pathway starting from isobutyryl phloroglucinol, is synthesized in translucent essential oil bearing glands and hereby stored as a consequence of its lipophilic isoprenoid moieties [10,92]. 

Secretatory tissues, despite being present in the whole plant, are mostly located in specific organs like leaves and in particular in reproductive parts of SJW. For example, it is well-known that hypericin content in whole plants increases during the phenological cycle and reaches its highest level (2.37 mg/g fresh weight) at full flowering, usually occurring after 52 growth days followed by a decrease [39]. Reproductive parts house the highest level of hypericins, while stems are the organs with lower amounts (0.2-0.3 mg/g dry weight approx.), followed by mature fruits (0.9 mg/g dry weight approx.), floral buds (0.99 mg/g dry weight approx.), unripe fruits (1.49 mg/g dry weight approx.) and flowers in bloom (2.37 mg/g dry weight approx.) and the highest amount is found in the upper three nodes of the plant [93]. As a consequence of such localization, a gradient of accumulation of hypericins is thus expected during the ontogenetic development of SJW and during the seasons of the year. Usually, optimal hypericins content coincide with the latter stages of anthesis, when inflorescences consist especially of open flowers. Collecting later in the flowering period, as opposed to earlier when inflorescences are mainly composed of buds, may be additionally desirable because of the greater concentration of hypericin and pseudohypericin in the floral tissue that makes up a substantial part of the harvested biomass. Dianthrones like hypericin and pseudohypericin increase with the development of blossom buds, mainly because those substances are located mainly in stamina and petals. Dark glands in these organs, it must be noticed, are about twice in size if compared to leaves and petals, while those located in sepals are smaller [89]. As a consequence, hypericins concentration is about 15-20 times higher in stamens than in petals during the whole floral ontogenesis (Table 1). This accounts also for their net loss in fading flowers [94]. Such behavior was noted by various researchers, which reported a decrease in hypericin with the advancing of stages, whereas hyperforin increased during the end of the reproductive phase [95]. In cv. Topas, the following pattern for total hypericins was noted: 44.8-68.9 mg/g dry weight in yellow buds, 78.6-80.9 mg/g dry weight in flowers, 60.1-62.0 mg/g dry weight in fruiting flowers [96]. Hypericins are not transferred through the xylem sap and are very low in the whole xylematic tissues. During flower ontogenesis, hyperforin content increased constantly from 25 in young buds to 85 mg/g dry weight in unripe fruits, most likely as a consequence of its elective accumulation in the pistil [94,97,98]. During fruit ripening, the adhyperforin content can increase from 9 (flowers) to 19 mg/g dry weight (capsules) [98]. On the contrary flavonoids and naphtodianthrones are biosynthesized and accumulated in dehiscent parts of the flower (sepals, stamen, petals). On the contrary phloroglucinols like hyperforin remain stable during flowering, but increase their presence in unripe fruits, which should be thus preferably collected if a maximization of this chemical is desired (Table 1). The accumulation of hyperforin in the pistil and in unripe fruits is likely a consequence of its accumulation in chloroplast-containing tissues, where translucent glands are more abundant [92]. Populations and breeding lines with a late flowering have proven to provide higher productivity in terms of biomass [6].

The flavonoid content as well undergoes fluctuations through the vegetative stages of SJW, as monoflavonoids are at their maximum before blossom, while biflavonoids provide maximum results at bloom. In particular, biapigenin and hyperoside both increase in the developing buds and drastically decrease during overblooming [100]. Quercitrin and isoquecitrin reach instead their maximum in unripe buds. Other phenolics like quercitrin and quercetin in particular show an upward trend towards floral ontogenesis, while the contrary is reported for chlorogenic acid, hyperoside and apigenine derivatives [101]. Rutin seem to be higher in leaves and during vegetative stages than in reproductive tissues, even if a slight rutin increase has been noted during fruit development [102]. In fact, rutin, hyperoside and isoquercitrin seem to be more abundant in photosynthetic tissues (mono-biflavonoids ratio 1:0.03), while quercitrin, quercetin, biapigenin and amentoflavone are mainly secreted in flowers (mono-biflavonoids ratio 1: 0.15) [103]. Pluhar *et al*. reported that the highest hypericins content (4-16 mg/g dry weight) has been obtained in the second year of cultivation, while the amount of flavonoids was maximum in the third year (24-30 mg/g dry weight) [7]. 

### 3.1. Ontogenetic stage

The histochemical localization of various phenolic compounds isolated from the phytocomplex seem to be somewhat species- and stage-specific. In fact, secretory tissues (both nodules, translucent glands and canals) are not present in all species of the genus nor at every stage of the developmental cycle [104]. Their presence and frequency is significantly variable among plant organs and during ontogenesis the different polyphenols biosynthesized by SJW provide a different pattern [105]. Hypericins and acylphloroglucinols have been detected in seedling yet at early stage of development and despite the fact that inflorescences are considered richest in nodules, the best sites for the extraction of the secondary metabolites and time for their collection must be choosen according to the chemicals desired: while flavonoids (as a sum of mono and biflavonoids) peak at flowering bloom, the amount of hyperforin keep increasing during fruit ripening [38,58,94]. The content in hypericin and other dianthrones, instead, reach its maximum in floral buds just opened, then suddenly drops to almost zero during fruit ripening. Such behavior is most likely due to a different localization of secondary metabolites: hypericin and pseudohypericins are located mainly in the petals and in the stamens, which can justify their decrease in older, wilting flowers [53]. Similarly, the dramatic decrease in flavonoid content after anthesis seem to be a consequence of their localization in sepals and petals. Flavonoid pattern, in fact, show an increase from small buds to overblown flowers, mainly due to the net increase in quercitrin in the latter. Biapigenin and biflavonoids, mainly as a consequence of the continuous loss of the tissues where they are mainly located (pollen and stamen), show an increase during bud development and a subsequent continuous decrease during blooming. Chlorogenic acid and hyperoside are also at their maximum in leaves during full flowering stage [101]. The flowering, however, is extremely quick and this must be taken into account in order to optimize the quality of the collected material. Buds grows quickly and their average development from greenish to deep yellow is 5.5 days [6]. Flowers open at sunrise and wilt already at noon of the second day if weather is cool and wet. Under warm-temperate climates yet on the first day evening they could show necroses and first signs of wilting: the window for collecting flowers is thus very narrow. The entire flowering phase of an individual lasts about 3-4 weeks and different ratios of fresh to wilting flowers may affect the phytochemical profile. Collecting plants at the same time of the day may be advisable, in order to minimize differences in such ratio.

Hypericins have been detected already at the plantlet stage, regardless of the cultivar employed [65]. An amount in the 90-100% range of hypericins is comprised in the 20-30 cm upper part of the plant during flowering stage, but it must be remarked that also plant height suffer of high variability and the flowering segment may fluctuate from 16 to 43 cm and subsequently affect dry matter yield [33,106,107]. Similarly, from plants collected in two different years, an almost double pseudohypericin content in drug obtained from flowers instead of 5-cm-stem tops (0.44-0.71 and 0.71-1.52 mg/g dry weight) and hypericins precursors are seemingly following the same hypericin trend during flower and fruit development [67,96]. The approximate ratio of the amount of hypericin in flowers, leaves and stem is approximately 30:10:1. Despite some observations on *Hypericum* species suggested a possible diurnal fluctuation on hypericins within the whole genus, the daily variation of total hypericins was evaluated in some wild Turkish accessions of *H. perforatum* [93] and no diurnal fluctuation in the overall hypericin content was observed. This seems to be in line with the absence of translocation pathway for such compounds within different plants’ organs. Similar results was reported for total phenolic content which also provided the highest results in reproductive parts at floral budding and in leaves at fresh fruiting stages [108]. However, on leaf samples hypericin reached its maximum at night during the blooming stage (1.43 mg/g dry weight dry weight) [93]. It must be noticed that the same authors reported differences within single organs during the daily cycle. For example, hypericin amount in floral buddings was almost halved from dawn until the evening while secretion in leaves increased during the same time span. A possible loss due to photo-oxydation could be a cause. On the contrary it increased both in full flowering and fresh fruiting stages. 

During the first year of cultivation biomass yield of flowering tops is usually low (7-30 g/plant, average 15), but can increase considerably during second year (43-119 g/pl, average 34) and remain high during the third (13-134 g/pl, average 33). This remark intrinsically possess elements of obviousness, as SJW plants reach their final size and morphology in the second year of growth [33]. During the same time span, however, hypericins content slightly decrease while hyperforin remained stable [7,33,59,96]. It seem possible to perform more than a single cutting without a harmful effect on the harvested plant material even if some authors report a lower hypericin content in the second cut while others reports the contrary [7,59,106]. Differences could be accounted to different soil properties and/or fertilization, which were unfortunately not reported.

### 3.2. Seasonal variation

Studies on the seasonal variation of hypericins are limited to observations performed under Australian climate conditions [71]. As expected, during spring growth and flower development, the concentration of hypericins increase rapidly to peak in early summer when flowering had almost finished and fruiting capsules were forming (2-4.8 mg/g dry weight). Total hypericins then decrease as flowers are lost and capsules are maturing over summer. Autumn saw a steady decline to the winter minimum levels (0.02-0.2 mg/g dry weight). Finally, as noticed by some authors, protohypericins, which can be converted into hypericins by sunlight exposure, are more abundant in plants collected in june rather than in september, resulting in a better drug and suggesting earlier summer collection as a better choice [109,110]. An early collection date seem to be favourable, even if two harvests per year are planned [33].

### 3.3. Future developments

According to the informations available in the literature, the choice of the correct developmental stage and of the appropriate harvesting time seems mandatory to maximize phenolic content and obtain an optimal phytochemical profile (hypericins and/or hyperforin content) for *H. perforatum* plant material aimed to the herbal and pharmaceutical market. A further problem in SJW production is however introduced: above mentioned metabolites do not peak at the same developmental stage, so that in most cases a radical choice (i.e. go for an high amount of a single substance) or a compromise must be made. For example, it may be suggested that SJW should be harvested when fruits are ripening to guarantee the highest level of hyperforin, while flowering tops are better suited for their hypericin content. This could also be a reason for the great variability in hyperforin/hypericin ratio reported in the literature and makes the adequate interpretation of available data a hard task. 

On the whole, a great deal of work should be focused on the adjustment of hypericin and hyperforin content. Some reports on biosynthetic behavior of SJW during ontogenetic stages and seasons of the year are in fact somehow contradictory. It must be remembered that genetic, phenotypic and environmental differences between sampling sites and populations may occur easily and influence the final results. Furthermore, the rapidity of the anthesis and of floral wilting is a critical factor and may be the cause of the great variability in phytochemical content often reported, making results very hard to compare. A higher degree of uniformity in experimental design should be suggested, i.e. by choosing for such kinds of evaluations plants grown in greenhouse. Data available show that there is no correlation between hypericin and hyperforin contents (as expected as their synthesis and storage occur in very distinct secretive tissues). It is thus theoretically possible to breed or find accessions containing high levels of both those substances [59].

## 4. Agricultural practices ( irrigation, soil, fertilization)

As a consequence of the ecological niche it occupies, SJW is well adapted to a variety of temperate climates and soil types. *H. perforatum* readily thrives in the wild in disturbed habitats that include roadsides, grasslands, overgrazed pastures or open shrublands and brushwoods, also in full sun and poor soils (both siliceous, clayey, chalky), where competition by native or forage species is very limited. Its capability to tolerate temperatures down to –15°C allow SJW to colonize habitats from 0 up to 1,600 m a.s.l. During the last decades *H. perforatum* cultivation has expanded, mostly under temperate climates, to provide the herbal market with an adequate amount of material, avoid the excessive variability intrinsic in wildly collected plants and reduce costs by means of mechanized harvesting. In fact, cultivated SJW grown at appropriate agronomic conditions could yield extracts with a higher amount of bioactive polyphenols and hence provide a higher market and therapeutic value. Different factors pertinent with field cultivation can influence both biomass production and phytochemical profile of medicinal plants: nitrogen and nutrient supply, mulching, irrigation, light exposure, temperature, intercropping, density, cultural methods. Some of them have been evaluated, although a complete definition of the optimal practices for such crop has not been defined.

### 4.1. Water availability

Flower top production seem to be enhanced (up to three times) by cultivation on a properly irrigated sandy soil, rather than silty soil [69]. Such differences on quantitative production of reproductive parts, however, do not necessarily extend to secondary metabolite yield and profile. Summer rainfall, instead, seem to determine a steady increase in hypericins biosynthesis and summer drought do not influence the production of flower buds [111]. To confirm such evidence, during a particularly rainy and cloudy year a higher biosynthesis of hypericins has been reported, suggesting that dianthrones -differently from other secondary metabolites like terpenes- may not be enhanced by water stress [71]. Water availability has been further investigated and found to be a significant factor in hyperforin biosynthesis, which share with terpenes part of the biosynthetic pathway. Leaves from greenhouse-grown plants submitted to a minimal vital irrigation (50 mL/die) were found to produce after 62 days an amount of hyperforin 3–4-fold higher than 1–2 years old individuals [112,113]. On the contrary, hypericins were significantly reduced by water stress, thus confirming the previous findings [71]. Different behavior may be related to the different physiological significance of hypericins and hyperforins and to distinct biosynthetic pathways. While in fact the latter may serve as an antioxidant support to overcame the oxidative burst subsequent to water stress, the biosynthesis of the former is most likely reduced as a consequence of the low carbon production by the process of photosynthesis during a prolonged drought. The length of water stress, however, is a further factor to be taken account of. Previous studies were limited to a six day time span and revealed an inverse reaction: a small increase in hypericins and a small decrease in hyperforin, joined with a net loss in flower dry weight. The net result of a brief water stress is considered negative in terms of value of the drug obtained: the small increase in phytochemical is outweighted by the biomass loss [114]. Different behavior may be also a consequence of a different experimental design. 

### 4.2. Altitude

SJW is well adapted to altitudes up to 1,500-1,600 m a.s.l. and in Afghanistan it has been found even up to 3,600 m a.s.l. The content of rutin and quercitrin have been noticed to be correlated with the altitude of the collection site: the former provided a negative correlation while the latter seem to be in positive correlation. The total flavonoid content has been reported to be positively correlated with altitude [56,94]. Rutin and hyperoside and in general flavonoidic content are also in positive correlation with altitude [55]. An exhaustive set of data on the effects of altitude on the accumulation of naphtodianthrones has been recently published, showing a remarkable increase in total hypericin content at high altitude in different Hypericum species, including *H. perforatum* [115]. The authors suggested that this result may be a consequence of greater light exposure and increased UV-B irradiation and this hypothesis seems to be in accordance with data summarized in Section 4.4. No informations are available regarding the influence of altitude on secondary metabolites like and phloroglucinols.

### 4.3. Fertilization and nutrient supply

Nitrogen supply to *H. perforatum* growing plants has a profound impact on its phenolic profile. Growth in a low-nitrogen soil and sand culture resulted in elevated levels of hypericins (2–3-fold), while a soil supplemented with N induced a 3-fold decrease in total hypericin production in fresh material but did not affect the mean number of dark glands per leaf [116]. The same authors noticed that the relative ratio of hypericin and pseudohypericin was not affected by nitrogen supplementation. Only a moderate decrease in nitrogen supplementation is however advisable to avoid the induction of chlorosis and subsequent unwelcome reduction in biomass production. It must be however noticed that some contradictory reports are available on nitrogen supplementation. In fact, plants cultivated in Iran and fertilized with 250 kg/ha N and 100 kg/ha P increased the number of flowering stems per plant and hypericin content [117]. Further research, performed on plants supplemented with 150 kg/ha N and 100 kg/ha P evidenced an increase both in hypericin, hyperforin and flavonoidic content, thus a definitive statement on this regard is still yet to come and may be also dependant on the original soil composition [118]. The combined effect of light exposure and decreased nitrogen supplementation were also tested and proved to be additive and independent, with a continuous enhancement both of red glands number and hypericins in the 106-402 μmol m^2^ s^-1 ^range [119].

### 4.6. Future developments

An exhaustive optimization of agrotechnological methods and their correlation to phytochemical content of SJW is somehow lacking and should be encouraged. The increase in biomass and in polyphenolic biosynthesis is a challenge that should be pursued with a multifactorial approach. Interesting perspectives, from the cultivation side, seem to emerge from greenhouse habitats with controlled environment, where growth conditions can be “tailored” and finely tuned to plants’ needs. Economical considerations should be however deepened, to evaluate if higher biomass yield, enhanced secondary metabolite production and higher cultivation density (up to 15 times higher than in open field) can cover the costs of this kind of cultivation [120]. Obviously, such number of variables and effects may account, besides possible genetic differences, for the consistent variability in phytochemical profile from SJW from different geographic sites. Consequences of intercropping and plant density on secondary metabolite production have not been evaluated.

## 5. Biotic and abiotic stress

If one consider secondary metabolism as an integral part of the plant capacity to modify metabolic processes in order to thrive and grow in adverse conditions, it comes as no surprise that both biotic and abiotic stress can lead to variations in *H. perforatum* phytochemical profile. This is further evident when one conceives SJW as an invasive and ecologically aggressive species, able to face environmental challenges and adapt to disturbed habitats like grazing lands. In various systems, biotic and abiotic elicitors have been shown to modulate the rate of production of bioactive chemicals of SJW, or – on the converse – SJW is able to adequate in a plastic manner its secondary metabolism to external stimuli. This was evidenced in various papers and includes the response of the plant's polyphenolic biosynthetic system to herbivores, to different kinds of microbial attack and to consequences of heavy metals exposure. 

### 5.1. Metal contamination

Metal contamination can affect the secondary metabolism of medicinal plants, seriously affecting their quality, safety and efficacy [121,122] and SJW makes no exception. According to various reports [123,124,125,126] *Hypericum perforatum* is a fairly good accumulator of heavy metals, Cu^2+^ in particular, and this behavior should be examined, both from the safety side and from the possible consequences on secondary metabolism. As a rule of thumb, soils rich in Pb, Cd, Mn, Zn and Cu should be avoided as not suitable for SJW cultivation. Heavy metal content in SJW has been noticed to be extremely fluctuating upon the sites of collection [124]. A loss in seed germination has been noticed (from 63 to 58%) and a significant decreased abundance of hypericin and pseudohypericins (21- and 15-fold, respectively) in nickel-treated seedlings [127]. A complete inhibition of hyperforin biosynthesis was also noticed. Recently, it was observed that *H. perforatum* ssp. *angustifolium* hypocotiles treated with various concentrations (within the mM range) of Cr(VI) for seven days produced a marked increase of protopseudohypericin (+135/404%), hypericin (+25/38%) and pseudohypericin (+5/379%) biosynthesis [128]. Finally, an heavy influence by fluoride exposure on the plant growth parameters has been reported, but no determination on the secondary metabolism effects were conducted [129]. 

### 5.2. Pathogens and herbivores

The production of defensive and communication substances is one of the primary purposes of plant secondary metabolism and the main reason for the existence of many of what we consider active principles. Most compounds of pharmaceutical and herbal interest are *de facto* phytoalexins or phytoanticipins produced via polyphenolic biosynthetic pathways and their abundance in the crude drug can be directly dependent on the degree of exposure to pathogenic aggression, as part of the inducible plant defense response [130]. Anthrones and phloroglucynols are considered defensive ploys against herbivores, e.g. causing photosensitization of grazing animals following ingestion [131]. Hyperforins have good antimicrobial properties and thus are expected to vary to some extent according to the plant exposure to biotic stress [12]. Much work on this regard has been made by Sirvent, Gibson *et al*., confirming a direct relationship between hypericins abundance and exposure to insect feeders (*Chrysolina quadrigemina, Spilosoma virginica, Spilosoma congrua, Spodoptera exigua*) [131,132]. In particular a 30-100% increase was detected in plants challenged with few generalist larvas, while a loss was reported in case of major exposure and no significative alteration in the phytochemical profile were induced by mechanical wounding or feeding by specialist herbivores. Generalist larvae can grow only on organs lacking of dark glands, while specialists can circumvent the light-induced toxicity of hypericin and thrive on the whole plant [88]. Such reports could offer hints on the higher abundance of hypericins reported in vegetative material rather than in reproductive plant parts seldomly reported in the literature. 

Further work concerned the influence of exogenous chemical elicitors (the well known phytoalexin inducers methyl jasmonate and salicylic acid) and pathogenic elicitor (*Colletotrichum gloeosporoides*, chosen for its good degree of host-specificity with *H. perforatum*) [133]. Total hypericin levels increased as much as 3.3 times in control levels when treated with 200 μM methyl jasmonate for 14 days, while higher hyperforin concentrations were detected in plantlets treated with 1 mM salicylic acid or 50 μM methyl jasmonate. Plantlets growth and subsequently biomass production were however negatively affected by toxicity already at 200 μM methyl jasmonate. Overall, salicylic acid was found to be highly effective on hyperforin biosynthesis. Such behavior has been confirmed and studied in detail in a number of *in vitro* researches in which the capability of jasmonic derivatives to elicite flavonoidic and naphtodianthrone biosynthesis to the detriment of anthocyanin accumulation in SJW was observed [91]. The induction of hypericins via inoculation of *C. gloeosporoides* resulted in a 2-fold incrementation if compared to the control at 1 x 104 spores/mL, while higher spores concentrations were detrimental to the plant health. An earlier report, however, noticed that both healthy controls and *C. gloeosporoides* infected plants contained the same levels of hypericins [52]. Recently, both *in vivo* and *in vitro,* an increased xanthone biosynthesis was noticed in plants exposed to *C. gloeosporoides* [133]. The elicitation of hypericin biosynthesis by fungal exposure is somehow supported by various in vitro findings on undifferentiated SJW cells [134]. SJW has been reportedly affected also by viral diseases, evidencing a lower amount of total methanolic extract and a statistically significant lower content of hypericins in cv. Topas, whereas cvs Hyperiflor and Hyperimed suffered minor and not significant alterations, with the sole exception of the increased abundance of hyperforin in infected “Hyperimed” [135]. The treatment with conventional herbicides can provide a slight augment in hypericin content [136]. Also *Phytophthora capsici* and *Diploceras hypericinum* spores were found to be able to modulate hypericin biosynthetic pathway, eliciting an increase in hypericin biosynthesis [137].

Finally, *Hypericum perforatum* has been reportedly frequently affected by wilt disease caused by phytoplasmas and such pathology induced variations on secondary metabolism [139]. Quali-quantitative phytochemical variations were in fact observed in dried flowering tops of cultivated *Hypericum perforatum* L. cv. Zorzi infected by phytoplasmas of the “ash yellows” class. The affected plants exhibited decreased amounts of rutin (1.96 vs. 4.96 mg/g dry weight), hyperoside (2.38 vs. 3.04 mg/g dry weight), isoquercitrin (1.47 vs. 3.50 mg/g dry weight), amentoflavone (0.12 vs. 0.39 mg/g dry weight), pseudohypericin (1.41 vs. 2.29 mg/g dry weight), whereas chlorogenic acid content was doubled (1.56 vs. 0.77 mg/g dry weight) [139]. Hypericin, quercitrin and quercetin contents were not severely affected. Host resistance may develop specific variations in the secondary metabolism of challenged tissues, inhibiting the flavonoid biosynthetic pathway and increasing the biosynthesis of caffeic and cinnamic derivatives [140]. The net results provided would fit with these hypotheses. The information at present available allow to state that hypericins and hyperforins are not phytoalexins but rather phytoanticipins (secondary metabolites that are already present in low quantities in healthy plant tissues and are induced by biotic or abiotic challenges). 

### 5.3. Light and Carbon availability

Environmental factors like light and CO_2_ supplementation as influencers of photosynthetic rate and thus of polyphenol production were evaluated in plants grown at different Photosynthetic Photon Flux Density (PPFD) providing interesting results [120]. Leaves from individuals grown in controlled habitat at 660 μmol m^2^ s^-1 ^PPFD and 1500 μmol mol^-1^ provided a 30 and 41 times higher content of hypericin and pseudohypericin than open field grown plants, respectively. A similar result has been reported regarding dark glands number in leaves at vegetative stage (with a peak at 550 PPFD) but with limited impact during reproductive stage (12 glands/cm^2^ instead of 18) [89]. Researchers evidenced that hypericins content is directly related both to CO_2_ concentration in the environment and PPFD; fresh and dry matter production were also enhanced in a similar way (+30%). Such effects should be considered as the consequence of an enhanced photosynthetic rate. No data, however, were provided regarding consequences on flower buds production and composition and despite being extremely appealing such approach seems to need further fine tuning before being evaluated for large-scale application. According to a different report, an increased light exposure resulted in a linear rise in the level of leaf hypericins. The increased light exposure translated also into a parallel increase in the number of dark glands and this was also somewhat confirmed by open-field experiments in which shade lowered the content of hypericin [53,119]. Under controlled environment, SJW plants grown under red light (600-700 nm) provided a dry weight higher than plants grown under blue lights, but no details on phytochemical consequences have been made to date [141]. It must be also pointed out that samples with the highest amount of hyperforin and hypericin ever collected in the wild were from an Armenian area with more than 300 sunny days per year [65]. Such behavior could be linked to the enhancement of the conversion of protohypericins into hypericins provided by the light. The photosynthetic rate was found to be linearly related both to dark gland number and hypericins accumulation only during the first 6 weeks of the plant’s vegetative stage.

### 5.4. Temperature

SJW plants have a limited capacity to react and tolerate high temperatures and this entails both into a loss in dry weight and in alterations in the phytochemical pattern. In fact, temperature have been found to be a significant factor, with plants cultivated at 25°C presenting a 25% hyperforin/hypericins ratio and a pseudohypericin/hypericin ratio 20% higher than plants grown at 30°. Such variation provide a further indication of the possible consequences of small environmental changes (i.e. climatic and geographic) on SJW phenolic secondary metabolism and may at least in part justify the great heterogeneity described in Chapter 1. Temperature, however, did not provide effects on phytochemicals concentration ratio of flower buds and shoots [39]. A gradual two-week increase in temperature (8-18-28°C) determined an increase in hypericin biosynthesis in plants cultivated in Canada [52]. Temperature stress, both positive and negative, administered few days before collection, may induce an inferior plant growth due to lower photosynthetic efficiency, while the decreasing temperature (from 35° to 15°C) determine a loss in hypericin and hyperforin biosynthesis in shoots grown under controlled environment. No exhaustive data on flowers and floral buds are available on this regard, however temperature in the high 20^s^ °C seem to be better suited for optimal biosynthesis of secondary metabolites, with better performances for hypericins and worst for pseudohypericin and hyperforin in such organs at temperatures above 20°C [113] .

### 5.3. Future developments

Despite their value towards a better comprehension of the mechanisms underlying the biosynthesis of hypericins and hyperforins and their ecological role, the above mentioned reports have limited on-field relapses. This is mainly a consequence of the extensive and significant loss in biomass production induced by biotic and abiotic stress. However, taking into account that most of SJW cultivation is organic, the consequences of *in vivo* exposure to pathogens must be deepened, while most of the available data refers on this regard to *in vitro* experiments. Being some of the most pharmaceutically important secondary metabolites of SJW polyphenols secreted in specialized tissues like red and dark glands, the biosynthetic behaviour of undifferentiated cells may however greatly differ from the consequences triggered by elicitation of the whole plant in a dynamic environment and thus may provide only limited knowledge in terms of production [142]. 

## 6. Post-harvest and stability

The variability in chemical composition of *H. perforatum* do not stop at the *in vivo* level. Various papers in fact report the instability of SJW polyphenolic phytocomplex during the post-harvest, handling, storage and marketing stages as a consequence of both light exposure, pH and temperature [99,143,144,145]. Post-harvest handling of plant drugs in the stages immediately subsequent the collection, in particular, may be relevant for consequences on the market value of the crude drug [146]. Instability of crude extracts, despite being related to similar factors, will not be considered here due to their pertinence to pharmacy and drug delivery.

Post-harvest light abundance and quality were evaluated on field grown plants, evidencing that several hours of exposure to light of freshly collected SJW do not produce any loss of hypericins, but modifies the protopigments/pigments ratio (protohypericin vs. pseudohypericin and hypericin), with a decrement in protopigments with the increase of sunlight exposure [110]. This suggests that drying flowers under sunlight may stop the conversion of protopigments into pigments faster than in buds (which are less penetrable by light) and that the presence of protective substances like flavonoids could similarly limitate such conversion. A 16 hour light exposure of flowering tops should induce a 20% conversion from protohypericin and protopseudohypericin to their stable forms. Previous research reported that the conversion of protopigments into pigments could be obtained after 4.5 h of sunlight exposure or 30 min. of artificial white light [147,148]. An extensive and detailed survey of the modification of SJW herbal drug appearance and composition during postharvest handling is available [149]. Both temperature and time were taken into account and useful determinations on the respiration rate of freshly cut *H. perforatum* cv. Topaz were made. Results suggested the drying of plant material at 10°C for up to 70 h, 20°C for up to 60 h and 30°C for up to 30 h to avoid an excessive decrease of visual quality of the drug (discoloring, hardening, wilting, fading). The authors also determined the need for an intermitted (30-50% of the day) ventilation of the freshly harvested material with cool air (e.g. night-time outdoor air) and not warm air, as a consequence of its unusually high respiration rate. The phytochemical quality of the drug remained fairly stable, with a reduction in hypericin and total flavonoid content of 20%. A remarkable 80% increase in hypericins and a 50-60% increase in flavonoids was however noticed at all three temperature conditions (10, 20, 30°C) in material obtained from younger plants (first cut of the first cultivation year). Those increases are most likely to be accounted for a new synthesis physiologically occurring in younger and thus more vital tissues, as reported for other medicinal plants. More recently the consequences of drying temperature and freezing on hypericin and flavonoid content were monitored in Brazilian samples, noticing that freezing can be detrimental to the hypericin (whose abundance was almost halved in samples frozen in liquid N_2_) but not to the flavonoid content [150]. Best drying results, from the standpoint of hypericin and overall flavonoid content, were obtained at 50°C. Economical evaluations on such behavior might be of great value to assess the possible advantages of early collection and appropriate handling or industrial processing, as a shorter cultivation time could lower the costs of production. The consequences of air-drying at room temperature on the phytocomplex was also evaluated, evidencing a two-third loss in flavonoid content and no statistical differences in hypericin [151]. This behavior, if compared to previous reports, is most likely due to the higher drying temperature. Hyperforin content evidenced a drastic loss from 109.5 mg/g fresh weight to 6 mg/g in air-dried samples sunlight-exposed and from 101 mg/g fresh weight to 5 mg/g in air-dried samples extracted in the dark, suggesting that to obtain extracts rich in such compound room temperature air-drying is not suitable and light exposure must be avoided. As a rule of thumb, hyperforin-rich extracts should be obtained from fresh and not dried material. This could be also due to a faster degradation of translucent glands, which are just under the epidermid and more prone to damage due to evaporation and heat [6]. An early harvest at a late bud stage is advisable in order to maintain the highest hypericin content after drying, while a late harvest is favorable to avoid excessive losses in hyperforin. Within the range of 40-80°C biapigenins, hypericins and hyperforins are not dramatically influenced by temperature, while flavone gycosides, rutin and hyperoside can undergo a marked decrease. Many papers have however described the formation of degradation products in crude extracts of *hyperici herba* mainly due to light exposure [99,143,144,145], thus the above mentioned treatments must be conducted under a strict phytochemical monitoring.

### 6.1. Future developments

No data are presently available on the consequences on polyphenol content of post-harvest treatment of fresh or dried SJW drug with ethylene, controlled or modified atmosphere conservation, gamma-irradiation, freeze drying, artificial drying, spray drying, microwave drying and their large scale economics. The same applies to long-term storage of the crude drug which has been instead deepened for other high-selling botanical drugs like Echinacea and Ginkgo [152]. In summary, most of the literature on postharvest degradation of the main secondary metabolites of SJW is focused on the extract and the actual knowledge on the crude drug is limited and should be deepened for three main reasons: 1) a correct drying and an adequate light exposure time of freshly collected plant material could enhance the polyphenolic abundance in the final drug; 2) different handling of the crude material could lead to scattered and unreliable reports in literature (also in terms of polyphenolic profile and pharmacological equivalence) and; 3) the biological activity and toxicology of the degradation products are not known and their presence must be minimized.

## 7. Conclusions

Pharmaceutical, therapeutic and commercial quality of SJW derived products is strictly dependent on the intrinsic characteristics of the starting herbal material and commercially available products have proved to be extremely fluctuating. The hypericins content of commercial SJW preparations, for example, may differ between 3 and 114 % from the amount claimed by the producer and the same applies for hyperforin [49,121,151]. It is clear that the fundamental prerequisite for the supply of SJW drug with equally high quality and efficacy is the breeding of homogeneous varieties with an high polyphenolic content and their cultivation under controlled conditions. The selection of such varieties and the definition of the optimal conditions is still ongoing, although some hints are evident and some blanks have been filled during the last decade. The development of Good Agricultural Practices focused on secondary metabolism and covering both SJW cultivation, harvest and post-harvest influence on phenolic content should to be considered pivotal in order to provide the growers with a viable reference for long-term profitable cultivation. Scattered actions distinctive of niche cultivations, where impromptu or improvising mode of action often are praxis, must be avoided. Such process is far from being accomplished, but some indications and suggestions can be drawn and some deficiencies can be pointed out and summarized. 

### 7.1. Focusing breeding and agronomic practices on phenolic metabolism

In order to satisfy the market demand, breeding lines with specific, definite and reproducible profiles of active principles and agronomic characters are needed. Varieties currently most widely cultivated (Topas, Hyperimed, Hyperixtract) do not seem to always coincidentally fulfill market requirements in terms of active principles content and cultivation (amount of active polyphenols, resistence to pathogens, gross yield) [68]. Some accessions of SJW have been therefore selected during the last decades to improve germplasm, but selection criteria mainly concerned resistance and biomass production, while in just few cases the improvement of secondary metabolism efficiency was properly taken into account [59]. Such goal, in the depicted overview, is being accomplished at the present time by two different approaches: biodiversity screening and phytochemical-guided in-field selection. Natural populations and breeding lines of SJW show in fact very high variability in terms of phytochemical profile and wild accessions diversity can constitute an useful reservoir of starting material. Unfortunately, a complete screening of hypericin and hyperforin content in wild SJW is far from being completed. Genetic factors strongly affect both plant yield and bioactive substances biosynthesis in SJW, making the availability of elite germplasm a fundamental factor for field cultivation of superior quality herbal drugs based on *H. perforatum.* As a result of different climatic and geographic differences, each region may offer ecological conditions peculiar to itself and thus determine different phytochemical profiles in quantitative terms. Such modifications may occur even between nearby locations [61]. Some areas of the world where SJW grows have not been screened to evaluate wild local populations. Middle East, Western Siberia, China, North Africa, Canary Islands, South America are among them [6]. Genetic fingerprinting of the available accessions is not performed alongside with chemotype-driven fingerprinting, *de facto* making almost impossible to confirm unequivocally if the reported fluctuations are a consequence of phenotypic adaptation or of a different genetic trait. From a methodological standpoint, the most interesting development within this field would be the implementation of molecular techniques alongside with the more usual pharmacognostic and agronomic approaches. The development of DNA marker-assisted selection of desirable chemotypes will undoubtedly provide a considerable boost in germplasm selection of polypenolic and hypericin-rich lines. In other words, methods of DNA fingerprinting for SJW are becoming available [73,74,75,76,77,78,79,80]), but providing an adequate contribution to the field their application requires a stringent multidisciplinar approach that is often lacking [154]. This field of research, despite its capital relevance for SJW enhancement is yet unexplored.

Selection of high-yielding hypericin lines depends on the genetic stability and on stable transmission of such genetic trait. On this regard the fact that seed-propagated plants retain or enhance hypericin content allows the breeders to act in a traditional way, but on the other side the eventual hybrid production and the facultative apomixis can contribute to obtain mixed and variable results, in wild material in particular. Interspecific hybridization has been recently reported but its consequences on the polyphenolic profile of SJW has not been evaluated so far. On this regard some RAPD based methods to precisely verify intraspecific crossings during hybrid selection are becoming available and could be helpful during the breeding process [155]. Comparative breeding and long-term selection of lines evaluated not simply for their agronomic advantages (gross yield, resistance, etc.) but also for their phytochemical profile and content must be pursued. A synergistic effort between agronomists and phytochemists must be encouraged on this regard. 

### 7.2. Standardized quantifications

Care must be taken in experimental design of breeding selection and chemotaxonomic surveys; the amount of phenolic metabolites being strictly dependent on the development stage of flowers and being anthesis and flower life particularly short, plants must be harvested at approximately the same maturity level in order to avoid differences and discrepancies due to different growth stages. Data presented are often quite discrepant, also as a consequence of different experimental approaches. The disparity of metabolite concentration in published data could be to a great extent caused by a mismatch of the blossoming stage or extraction method and, in general, by inadequate post-harvesting processing. As previously remarked, a higher degree of uniformity in experimental design, analytic procedure, sample handling must be considered a priority to obtain reproducible data whose utility should be as widespread as possible. Regarding the evaluation of different and huge numbers of batches of cultivation and to solve the problem of specific phytochemical tracking provided by HPLC methods, the application of multivariate data analysis and NMR metabolic fingerprinting (possibly with quantitative data) is becoming more popular and could be suggested for fast screening and discrimination of great numbers of data on cultivars and breeded lines [156,157]. In fact, such approach allows the simultaneous tracking of different phytochemical parameters on all relevant chemical classes of SJW phytocomplex. This is of particular relevance due to the different phenolic substances reputed to contribute to the antidepressant activity of SJW (flavonoids, hypericins, hyperforins) and to its possible side-effects [158,159]. Because of the uncertainties regarding the compounds responsible of the pharmacological activity of SJW, the widest range possible of phytochemical differences should be screened, taking into account all constituents at the same time. This would also help more properly the individuation of possible synergisms within major and minor constituents and the expression of pharmacological activities.

### 7.3. Standardized production

Besides intrinsic factors, the secondary metabolism of SJW appears to be clearly influenced by extrinsic factors, related to phenotypic adaptation and agronomic behavior. As described in chapter 5, total hypericins content can be increased by elicitation (biotic, abiotic, chemical) and this may lead to affirm that these metabolites are part of the inducible defensive system of SJW. The specific localization of hypericins in the androecium makes also harvesting and post-harvesting stages extremely important. The development of a collection methodology and drug processing (i.e. winnowing) aimed at reducing the loss of stamens will undoubtedly increase hypericin concentration in the final product; the collection of flowers just before the anthesis goes in such direction. The different responses provided by *H. perforatum* to different kinds of stimuli, in the meanwhile, suggest that its capability to biosynthesize bioactive phenolics could be selectively enhanced or inhibited in an independent manner. On this regard an increasing amount of knowledge is growing also on the possibility to produce a tailored *H. perforatum* phytocomplex, enriched in specific compounds or optimized for a specific aim, by means of bioreactors, hydoponic culture. Despite the limiting cost/efficacy ratio of this approach, the increasing yields obtained and the concurrent diminution of technology's prices may represent a good viaticum for a biotechnological production of SJW secondary metabolites competitive with other forms of cultivation [1,84,160,161,162,163]. Making use of extremely controlled environment systems seems to be a feasible way to standardize SJW cultivation and consistently increase quality. Furthermore, stability studies must be encouraged to optimize storage and harvesting conditions, with the aim to provide products as sure, predictable and efficient as possible. 

### 7.4. Provision of more knowledge about hyperforin and other bioactive constituents

The knowledge regarding the optimization of hyperforin biosynthesis seems to be rather defective, despite its importance in the expression of therapeutic properties of the drug. The conclusion that *H. perforatum* samples can be distinguished from all other *Hypericum* species by the concurrent presence of rutin and hyperforin has been proven false, although a limited number of species displays such a trait. Recently, hyperforin has been identified as the responsible of the induction of CYP3A and of the subsequent interference between SJW and drugs [21,164,165]. Being plant-drug interactions one of the main drawback of SJW long-term administration in mild-depression treatment, the selection of hyperforin-low cultivars may be an interesting option both from the commercial and the therapeutic point of view. On the contrary, its presence seem to be crucial to provide clinical activity of Hypericum preparations, highlighting the importance of a well balanced and controlled abundance of such substance in the phytocomplex [166].

For further in-depth considerations and developments, a major contribution from genetic, biochemical and biosynthetic studies should be expected. It would be in fact desirable to obtain the complete genetic coding for key enzymes involved in the biosynthesis of SJW’s main polyphenols and their regulating sequences in order to determine the overall genetic contribution to variability. At present time, in fact, only little information is available on this regard and also on the previous level: the complete biosynthetic pathway of hypericins and hyperforins is not yet completely elucidated neither have been adequately identified the rate-limiting step for precursor regulation nor the specific enzymes or intermediates involved [167]. The knowledge of the complete pathway and its regulations would allow researchers to directly and precisely act to enhance hypericins production yet from early stages of development.

In the context of selection and improvement of medicinal plants (from cropping up to the final user) an integrated approach in which competencies act synergistically and cutting edge technologies are applied to solve in field exigencies and the needs of the agroindustry is capital. If agronomy, biology and genetics used to be the go-to disciplines in crop improvement, to increment polyphenol biosynthesis in plants like SJW there is a clear need to develop strategies for breeding in strict cooperation with pharmacognosy and phytochemistry. Such approach would also benefit the knowledge and the development of cultivation of other best-selling herbal drugs like echinacea, ginseng or valerian.

*Sample Availability*: Not available.
